# Differences in boundary behavior in the 3D vertex and Voronoi models

**DOI:** 10.1371/journal.pcbi.1011724

**Published:** 2024-01-05

**Authors:** Elizabeth Lawson-Keister, Tao Zhang, Fatemeh Nazari, François Fagotto, M. Lisa Manning

**Affiliations:** 1 Department of Physics and BioInspired Syracuse, Syracuse University, Syracuse, New York, United States of America; 2 School of Chemistry and Chemical Engineering, Shanghai Jiao Tong University, Shanghai, China; 3 School of Biomedical Engineering, Ecole Centrale de Lille, Villeneuve-d’Ascq, France; 4 Centre de Recherche en Biologie cellulaire de Montpellier, University of Montpellier and CNRS, Montpellier, France; Universitat zu Koln, GERMANY

## Abstract

An important open question in the modeling of biological tissues is how to identify the right scale for coarse-graining, or equivalently, the right number of degrees of freedom. For confluent biological tissues, both vertex and Voronoi models, which differ only in their representation of the degrees of freedom, have effectively been used to predict behavior, including fluid-solid transitions and cell tissue compartmentalization, which are important for biological function. However, recent work in 2D has hinted that there may be differences between the two models in systems with heterotypic interfaces between two tissue types, and there is a burgeoning interest in 3D tissue models. Therefore, we compare the geometric structure and dynamic sorting behavior in mixtures of two cell types in both 3D vertex and Voronoi models. We find that while the cell shape indices exhibit similar trends in both models, the registration between cell centers and cell orientation at the boundary are significantly different between the two models. We demonstrate that these macroscopic differences are caused by changes to the cusp-like restoring forces introduced by the different representations of the degrees of freedom at the boundary, and that the Voronoi model is more strongly constrained by forces that are an artifact of the way the degrees of freedom are represented. This suggests that vertex models may be more appropriate for 3D simulations of tissues with heterotypic contacts.

## Author summary

Proper programming of the collective behavior of cells in biological tissues is required for successful embryonic development, and this programming breaks down in diseases such as cancer. Therefore, an important question is how to develop accurate computational models that can predict emergent collective behavior of cells. Two existing computational models, vertex and Voronoi models, have been used successfully to make quantitative predictions for collective cell behavior, and both predict the same fluid-to-solid transition in tissues, which is important for developmental processes such as body axis elongation. In this work, we study whether Voronoi and vertex models predict the same emergent behavior for cell sorting between two different tissue types, which is important for compartmentalization of tissues and organs during development. We find that the two models predict different features for the geometry and motion of cells, and that the more computationally intensive vertex model more accurately represents cell shapes and dynamics near an interface between two tissue types.

## 1 Introduction

Mechanical interactions between molecules, cells, and tissues are increasingly being identified as control mechanisms in development and disease. In order to make quantitative predictions about how physical forces impact processes such as tissue compartmentalization and cell migration in dense cellularized tissues, it is necessary to develop well-vetted mechanical models. In this work, we focus on models for confluent tissues, such as epithelial layers, where there are no gaps or overlaps between cells.

Traditionally, much of the computational work to simulate confluent tissue has been restricted to two-dimensional (2D) models [1–8]. While 2D models for confluent tissue are extremely powerful, they rely on several assumptions in order to approximate collections of cells that exist in three-dimensional (3D) space. One assumption is that all the cells of interest exist in a single monolayer, and that the mechanics of that monolayer is dominated by interactions in a single (usually apical) 2D plane. Another assumption is that any interactions with the environment, such as a basement membrane, can be approximated by body forces acting on cell centers or vertices. Finally, it is assumed that any fluctuations or dynamics in the unresolved third dimension (such as fluctuations in height) can be mapped in a simple way onto dynamics in the two-dimensional plane (such as fluctuations in cross-sectional area). But clearly, these assumptions are not always valid, and there are many systems that require a fully three-dimensional model such as in organ/organoid development [9, 10], cancer spheroids [11–13], and cell sorting in 3D cellular aggregates [4, 14].

In addition, compartmentalization of different tissue types and formation of boundaries between different cell types is crucial to proper function in development [15–22], and treatment of disease [23, 24]. While the exact mechanisms that drive compartmentalization and boundary formation are still an area of active research, several simple hypotheses have helped to drive forward the field over the past 60 years. The differential adhesion hypothesis (DAH) [25, 26] postulates that cell sorting is driven by differences in cell surface tensions, which in turn arise from differences in cell adhesion. This hypothesis correctly explains behavior in particle-based simulations of cells where particles interact with adhesion-like terms that depend on the distance between cell centers [27, 28].

In experimental observations of confluent tissues, where cells are not well-represented as sticky spheres, cell-cell adhesion must instead influence cell shapes and cell interfacial tensions. The differential interfacial tension hypothesis (DITH) [29] hypothesizes that differences in interfacial tension between two cell types drive cell sorting, and suggests that adhesion and surface contractility compete to create an effective interfacial tension between cells of opposite types, sometimes called a heterotypic interfacial tension [5, 30–32]. Importantly, DITH and DAH are not irreconcilable, as in many tissue types there are signaling feedbacks between adhesion and cortical tension [33] that lead to a down-regulation of cortical tension at interfaces with increased adhesion, so that both DITH and DAH predict similar outcomes [34, 35].

In confluent tissues, adhesion expression governs both individual cell shapes in a monolayer [3] *and* heterotypic interactions between two different cell types [30, 36]. It is not immediately obvious whether differences in cell shape alone are also sufficient to generate sorting. Recent experiments and simulations have suggested that at least in cell monolayers, differences in cell shape can generate small-scale segregation but not large-scale demiximing [3].

In contrast, simulations with heterotypic interfacial tension exhibit robust large-scale cell sorting [5] with sharp boundaries between tissue types, which closely mirrors the dynamics and boundaries observed in experimental tissues [37–40]. It is difficult to quantify heterotypic interfacial tensions directly *in situ*, although laser ablation approaches [41] can approximate differences in tension up to an assumed viscous constant. Alternatively, pipette aspiration experiments [35, 42, 43] can study tension properties of extracted cell doublets or triplets.

Recent simulation work has focused on understanding how the geometry of heterotypic interfaces impacts their mechanical properties. A study focused on 2D Voronoi and vertex models demonstrated that several different definitions of surface tension that are all equivalent in equilibrium fluids are not the same in confluent tissue models. Heterotypic surface tension along interfaces suppresses capillary waves, resulting in sharp yet deformable boundaries [31], with characteristic geometric signatures in cells near the interface [3]. Both effects appear to be less strong in vertex compared to Voronoi models in 2D. In 3D Voronoi model simulations, similar geometric signatures are observed including elongation of cells on the boundary, sharp interfaces, and compartmentalization of cells of opposite types [4].

Interestingly, a recent paper observes a small difference in the geometric behavior of cells on a heterotypic boundary in the 2D vertex and Voronoi models [4]. In the Voronoi model, cells across a heterotypic interface will align their cell centers, while in the 2D vertex model, their degree of alignment is significantly smaller. This presents a challenge to Voronoi models, as the choice of model degrees of freedom should not change predictions about tissue morphology if both models use the same energy functional and are meant to encapsulate the same physics.

Quite a lot of 3D modeling of confluent tissues has been performed using 3D Voronoi models [9, 44–46], mainly due to their computational efficiency and simplicity compared to 3D Vertex models. Almost two decades ago, a first 3D vertex model was described and simulated [47], and more recent work has explored other aspects of tissue mechanics with 3D vertex models [48–50]. However, this code was not made available open source, and there are significant subtleties and challenges in handling changes to topology in 3D vertex models, whereas those are handled automatically in Voronoi code bases. Very recently, Zhang and Schwarz have reported on rigidity transitions and topological protection in 3D vertex models for organoids, and concurrently released an open-source 3D vertex model code [10, 51], which permits a clear view of the coding choices made and is a significant contribution to the field.

Given the small difference seen between cell geometries in 2D vertex and Voronoi models, and the important role cell geometries play in both the physics of interfacial tension and in comparing model predictions to experimental observations, an important open question is whether there are any geometric differences near heterotypic interfaces in 3D vertex and Voronoi models. Therefore, we adapt the 3D vertex simulation code developed by [10] to investigate cell shape and dynamics at heterotypic interfaces. We compare that to the 3D Voronoi model to discern whether there are differences and, if so, explain the mechanisms that drive those differences.

## 2 Model and methods

### 2.1 Simulation methods

For both 3D vertex and Voronoi models, the simulated confluent tissue is composed of *N* = 1728 cells driven by active forces. Each cell *i* is represented by polyhedra with mechanics that are driven by the energy functional

$$\begin{array}{c} E=\sum_{i}^{N}K_{A}{\left(A_{i}-A_{0}\right)}^{2}+\sum_{i}^{N}K_{V}{\left(V_{i}-V_{0}\right)}^{2}+E_{0}, \end{array}$$ (1)

where the mechanical interaction forces between cells are given by *F*_*i*_ = −∇_*i*_*E*. The first term describes the competition between surface tension and adhesion where cell-cell contacts are represented by intercellular areas and have area modulus *K*_*A*_. The second term represents a soft constraint on the characteristic cell volume *V*_0_ with volumetric modulus *K*_*V*_. Here we are explicitly relaxing the constraint that cells are incompressible and suggesting that a combination of biological mechanisms, such as ion channels, regulate the volume to stay within a range parameterized by *K*_*V*_. Then both systems evolve under Brownian dynamics. The only difference between the two models is the degrees of freedom in the Voronoi model are the cell centers and the degrees of freedom in the vertex model are the cell vertices.

A three-dimensional non-dimensionalized shape index can be defined as $S_{0}=A_{0}/V_{0}^{2/3}$. As the shape index of a cell increases cells will become less circular or more elongated. Both models experience a rigidity transition in homogeneous systems in which the tissue goes from behaving solid-like to fluid-like as a function of the cell shape. In the 3D Voronoi model, this rigidity transition occurs at a cell shape of *s*_0_ = 5.413 and is identified by the point in which the shear modulus vanishes [52]. In the 3D Vertex model, the rigidity transition is identified by looking at the neighbor-overlap function and the tissue becomes fluid-like when the typical time-scale for rearrangements becomes zero which occurs at *s*_0_ = 5.39 ± 0.01 [10]. A similar agreement in the location of rigidity transition with cell shape is seen in the 2D vertex and Voronoi models [1, 2]. Unless otherwise noted, *S*_0_ = 5.6 in our simulations, so that the simulated tissues are in the fluid regime. This allows the tissue to efficiently explore configuration space to find low-energy states, and avoids additional surface stresses that arise between contacting solid surfaces.

While homogeneous tissue seems to behave quite similarly between the two models, it is also important to investigate tissue consisting of multiple cell types. As previously stated, the addition of an additional energy cost for heterotypic interfaces can cause large-scale demixing and compartmentalization between different cell types [3]. This additional interfacial tension also induces a large difference in cell morphologies for cells at the interface between the tissue types.

In the 2D vertex and Voronoi models the addition of interfacial tension between cells of different types changes the energy to

$$\begin{array}{c} E=\sum_{i}^{N}K_{P}{\left(P_{i}-P_{0}\right)}^{2}+\sum_{i}^{N}K_{A}{\left(A_{i}-A_{0}\right)}^{2}+E_{0}+\sum_{HIT}\gamma_{ij}l_{ij}, \end{array}$$ (2)

where the sum is over all heterotypic interfaces, *l*_*ij*_ is the interface between cell *i* and *j*, and *γ*_*ij*_ is the additional interfacial tension between cell types of cell *i* and *j*. While a seemingly small term, it turns out that this provides a remarkably strong collective effect. This term causes cells of different types to quickly and robustly completely demix [3, 30, 31] and can create sharp boundaries between cell types [31]. In completely confluent tissues these sharp boundaries generate constraints on the topology of neighbors.

First, for a vertex on a completely flat boundary to be stable, it must have four neighbors instead of the typical three-fold coordination. Since a stable vertex is under force balance and a flat interface will have two parallel edges, the third edge can never achieve force balance alone. This line of reasoning can be extended to prove that in homogeneous vertex models fourfold vertices are unstable [53, 54], although in Voronoi models stabilized fourfold coordinated vertices have been observed at very high shape values. In contrast, the addition of heterotypic interfacial tension can regularly stabilize four-fold coordinated interfaces in both vertex and Voronoi models at all shape values [31].

To investigate the configurations of cells on the boundary researchers quantified the distribution of edge lengths of cells on heterotypic interfaces in simulations of the 2D Voronoi model [31]. They find that there is an increasingly bimodal distribution of edge lengths as interfacial tension increases. This distribution is caused by perturbations to the stable interface due to finite temperature fluctuations. These perturbations will cause the predicted fourfold coordinated vertices to split and the single long-edge heterotypic interface will split into one large edge and one or two small edges.

In 2D Voronoi models, the breaking of these fourfold vertices generates a discontinuous restoring force that suppresses fluctuations. To quantify these discontinuities the researchers examined the restoring force generated by perturbing cells along a vector perpendicular to the interface. The authors then analytically calculated the restoring force on a Voronoi cell that is perturbed from a 9-cell square lattice. They found that the average restoring force cells experienced in simulations were very similar to that predicted by the analytic calculation and that the discontinuous restoring force scaled with the magnitude of the interfacial tension.

The inclusion of heterotypic interfacial tension in 3D models is similar to that of 2D except the additional edge cost is for the shared surface area between cells of different types rather than edges.

$$\begin{array}{c} E=\sum_{i}^{N}K_{A}{\left(A_{i}-A_{0}\right)}^{2}+\sum_{i}^{N}K_{V}{\left(V_{i}-V_{0}\right)}^{2}+E_{0}+\sum_{HIT}\sigma_{ij}A_{ij}, \end{array}$$ (3)

where the sum is over interfaces between cells of different types, *σ*_*ij*_ is the interfacial tension between the type of cell *i* and the type of cell *j* and *A*_*ij*_ is the interfacial area between cell *i* and cell *j*.

The authors of Ref [4] investigate the geometric and dynamic signatures that arise from this additional interfacial tension. There is rapid demixing between cells of different types which causes the tissue to compartmentalize. Both the speed and magnitude of this demixing are determined by the magnitude of the interfacial tension. In addition, the cell shapes on the interface will start to elongate and orient perpendicular to the interfacial axis. Additionally, as cell shapes on the boundary increase as interfacial tension is increased, cells in the bulk will round up and decrease their cell shape.

Cell orientation is calculated using the moment of inertia tensor of the best-fit ellipsoid to the cell vertices. Then the orientation is defined as the angle the long axis of the ellipse makes with the interface. The authors find that as heterotypic interfacial tension increases the cells go from random orientation as in the case with no heterotypic interfacial tension to highly oriented perpendicular to the axis of tension at high values of interfacial tension.

They also investigated a similar effect to what was seen in 2D which was the effect heterotypic interfacial tension had on the interfacial area along the interface. They observe a similar behavior to the 2D models, as the magnitude of the interfacial tension increase there is an increasingly bimodal distribution of small area facets and large area facets. This suggests a similar phenomenon to the breaking of fourfold vertices in 2D.

Finally, the authors noticed that cells on the boundary start to resemble one-sided prisms with flat interfaces at the boundary. In a Voronoi model to achieve this geometry, cells on one side of the interface would need to align their centers in a plane parallel to the interface. Additionally, cells across the boundary must align their cell centers to minimize the distance between their centers in the plane parallel to the interface such that the cell centers become stacked or registered. This registration effect is defined in a system in which the heterotypic interface is in the XY plane in the following equation.

$$\begin{array}{c} R=1-\frac{d}{l_{0}}, \end{array}$$ (4)

where *d* is the distance between neighbors across the interface in the XY plane and *l*_0_ is the average lattice spacing. If a cell is perfectly registered directly on top of its neighbor the registration will be unity and if they are perfectly misaligned half a lattice spacing away the registration will be zero. Sahu et al [4] find that as the interfacial tension magnitude increases, the height of cells on the same side of the boundary converges and that registration goes from roughly uniformly distributed to being highly peaked near unity.

In the paper supplement, they investigate this registration effect in the 2D models. They find that while the registration for the 2D Voronoi model shares similar behavior to the 3D Voronoi model, the 2D vertex model exhibits differences. The vertex model goes from a uniform registration to having a registration peak around a value of *R* ≈ 0.55, which is distinct from uniform but obviously less than the value near unity seen in Voronoi models. The authors hypothesize that the difference is due to extra degrees of freedom in the vertex model which allows a relaxation of some of the constraints at the interface. But this poses the question: are the geometric signatures seen in the 3D Voronoi model robust to the choice of model?

To investigate the differences between these two models in three dimensions we adapt the 3D Voronoi model code used in Ref [44] and the open-source 3D vertex model first published in Ref [10].

The equation of motion for both models assumes overdamped dynamics with the interaction forces given by the spatial derivative of the energy functional, Eq 3, with respect to the positional degrees of freedom. In addition, we include fluctuations as a translational white noise on each of the degrees of freedom—either the cell centers or vertices, respectively. This generates the following equation of motion:

$$\begin{array}{c} \frac{dr}{dt}=\mu \nabla E+\eta , \end{array}$$ (5)

where *η* is a white noise process with diffusion constant *μk*_*B*_*T*_*eff*_. In our simulations we set *μ* = 1, *V*_0_ = 1, and report lengths in units of the cell length $l=V_{0}^{1/3}=1$. Energies are reported in natural units of $K_{s}V_{0}^{4/3}$. In these natural units, our simulation time step is *dt* = 0.01. We tune the magnitude of the white noise in each models so that the mean-squared displacement of cells is diffusive and the same in both models at our chosen value of the shape parameter *S*_0_. In practice, we set the fluctuation magnitude in the Voronoi model to *k*_*B*_*T*_*Vor*_ = 0.1 and in the vertex model to *k*_*B*_*T*_*vert*_ = 0.1.

The simulation data is available at [55].

### 2.2 Experimental methods

To image the ectoderm-mesoderm boundary in the Xenopus gastrula, cryosections were prepared from Xenopus gastrulae expressing membrane-targeted GFP [56], and immunolabelled using an anti-GFP primary mouse antibody (Thermofisher/Invitrogen) and a secondary anti-rabbit Alexa488 antibody. *z* stacks were obtained by confocal microscopy using a 40x, NA = 1.25 oil objective. Maximal projections spanning 3–5*μm* thick slices were used for analysis. Outlines on cells on both sides of the boundary were manually segmented using polygon selection and ROI in ImageJ.

## Dryad DOI

10.5061/dryad.4tmpg4fgn [55].

## Results

### 3.1 Comparing vertex and Voronoi model structures in 3D

First, we investigated the behavior of a 3D vertex model simulation comprised of two different cell types with heterotypic interfacial tension between them. Just as seen in 3D Voronoi models [4], cells rapidly segregate and become completely demixed, with a snapshot of a demixed states in each model shown in Fig 1C and 1D. The speed and magnitude of this demixing increase as the magnitude of the interfacial tension is increased, as shown in Fig 1E. Additionally, if the tissue is initialized in a completely demixed state, the boundaries will remain stable and the tissue will stay demixed, as shown by the darker green and magenta lines in Fig 1F. This confirms that the demixed state is energetically preferred.

**Fig 1:**
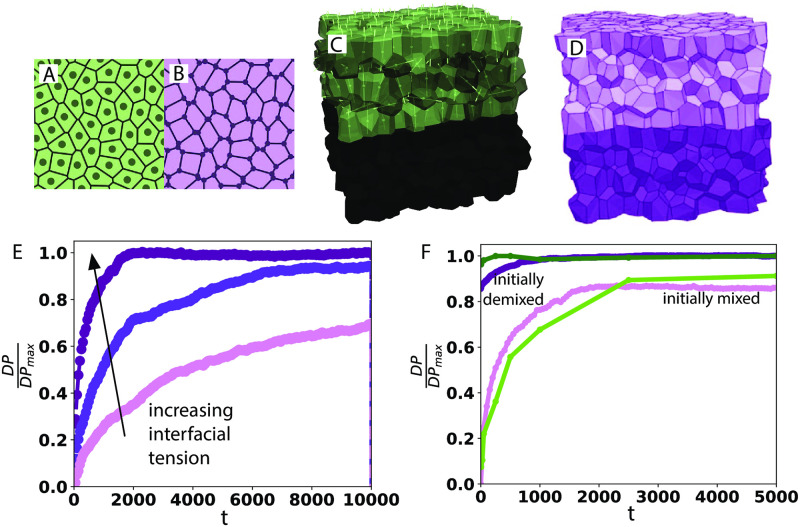
(A,B) Schematic of 2D Voronoi model (green, degrees of freedom are cell center) and 2D vertex model (purple, degrees of freedom are cell vertices). (C,D) Snapshot of the 3D Voronoi model (green) and vertex (purple) simulations with a heterotypic interface between light and dark cells. (E) Demixing behavior of the 3D Vertex model initialized in a mixed state with *DP* ≈ 0. The color represents different values of the heterotypic interfacial tension: *σ* = 0.1 (light pink), *σ* = 0.2 (medium purple), *σ* = 0.5 (dark magenta). (F) Demixing as a functional of initial conditions between 3D Vertex and Voronoi models. The dark green (Voronoi) and dark magenta (vertex) are initialized in a sorted state and remain sorted. The bright green (Voronoi) and light pink (vertex) are initialized in a mixed state and rapidly sort.

Next, cell shapes in the tissue were examined. A simulation snapshot of a smaller system highlighting individual cell shapes is shown in Fig 2A In the 3D Vertex model, the cells in the bulk of the tissue, shown as dashed lines in Fig 2B, decrease their observed cell shape index as the magnitude of the heterotypic surface tension increases, consistent with the behavior observed in the Voronoi model. Similarly, cells on the boundary, shown as solid lines in Fig 2B, experience increases in cell shape with heterotypic tension, and the magnitude of the increase is even larger than the Voronoi model at large values of interfacial tension. This is likely due to the extra degrees of freedom in the vertex model allowing cells access to a wider range of cell shapes.

**Fig 2:**
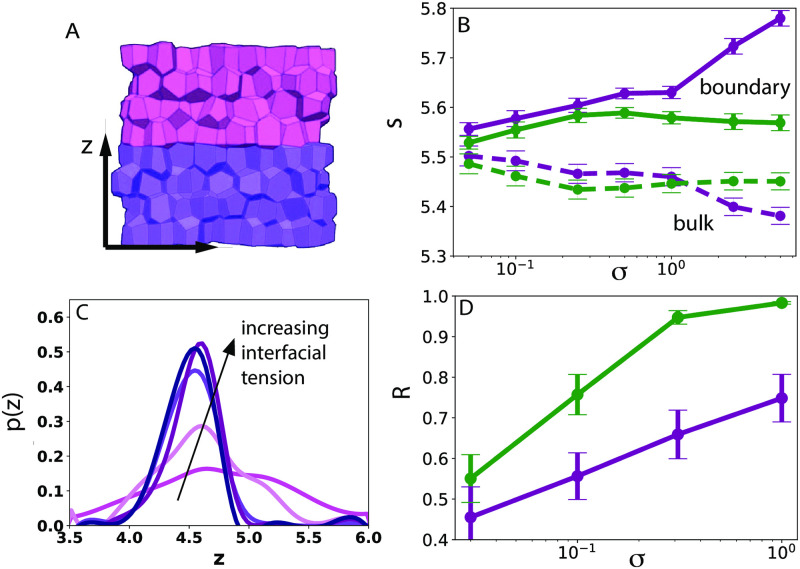
A) Zoomed simulation snapshot of the 3D Vertex model with heterotypic interfacial tension between cells of different types. B) Cell shape *s* as a function of the magnitude of heterotypic interfacial tension *σ* for cells that are adjacent to the boundary (solid lines) and cells that are in the bulk and do not share a heterotypic interface (dashed lines). In both models, the shape index of the boundary cells increases, while the shape index of the bulk cells decreases as a function of increasing interfacial tension. Error bars represent the standard error with respect to each ensemble. C) The probability distribution (pdf(*z*)) of the heights of cell centers (*z*) reported in natural length units from the bottom of the box. Different colors correspond to different magnitudes of the heterotypic interfacial tensions *σ* ∈ 0.04, 0.08, 0.16, 0.32, 0.64 from light pink to dark magenta. D) The registration of cell centers on either side of the interface (defined by Eq 4) increases for Voronoi (green) and vertex (magenta) models as a function of increasing interfacial tension. Error bars represent the standard error.

The cells along the boundary also exhibit similar registration behavior in both models. In the vertex model, cells on the same side of the interface will start to align their height in a plane as interfacial tension increases. Fig 2C shows a histogram of the heights (*z*) relative to the bottom of the periodic box. These cells will also start to register with cells across the interface with an increasing magnitude as heterotypic interfacial tension increases. The magnitude of registration is significantly higher in the Voronoi model than in the vertex model, shown in Fig 2D, and consistent with observations in the 2D models.

Moreover, the orientation cells on the boundary exhibit a surprising difference between the two models. In the Voronoi model, as the magnitude of interfacial tension increases the cells become highly oriented perpendicular to the interface. But, in the vertex model, the cells remain randomly oriented. To quantify this orientation effect over an ensemble we define an average orientation metric.

$$\begin{array}{c} \left\langle O\right\rangle =\left\langle \frac{{\left(\theta -\frac{\pi }{2}\right)}^{2}}{\frac{\pi^{2}}{4}}\right\rangle , \end{array}$$ (6)

where *θ* is the angle the long axis of a cell’s moment of inertia tensor makes with the heterotypic interface. This metric is designed such that the alignment of all cells completely parallel to the interface yields an average orientation of zero and alignment completely perpendicular yields a value of unity, with examples from simulations shown in Fig 3 panels (A-C). Random orientations correspond to 0.2 < 〈*O*〉 < 0.25. As shown in Fig 3(D), the cells at the boundary in both models are randomly oriented with 0.2 < 〈*O*〉 < 0.25 for very small values of the heterotypic tension, as expected. Strikingly, as the heterotypic interfacial tension increases, this metric displays a sharp difference between the two models. The average orientation increases steadily for the Voronoi model as heterotypic interfacial tension increases, meaning the long axis of the boundary cells becomes increasingly oriented perpendicular to the interface. In contrast, there is a negligible change in the orientation of vertex model cells.

**Fig 3:**
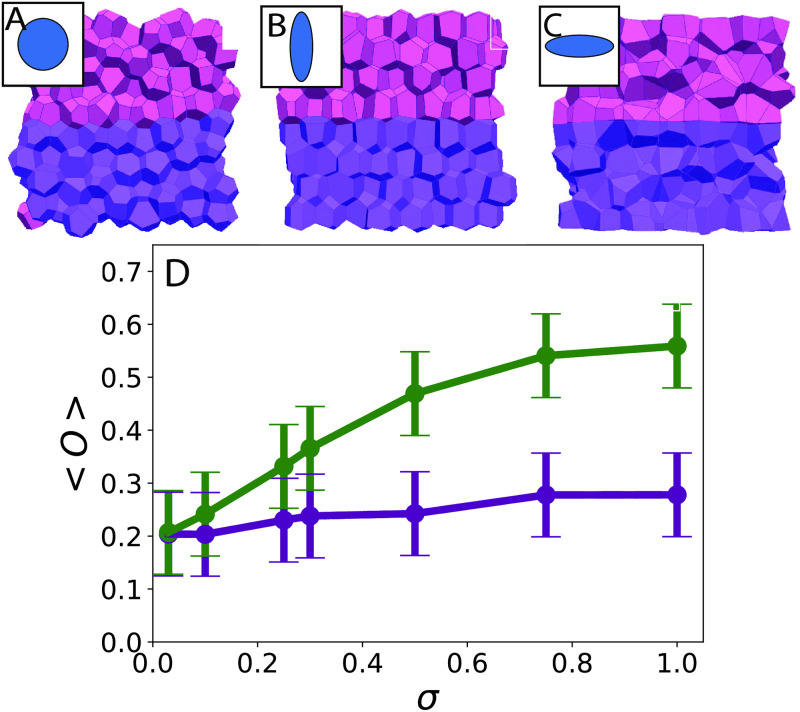
(A-C) Simulation snapshots of tissues with different values of average orientation 〈*O*〉 defined by Eq 6 (0.24, 0.38, 0.18 for A,B,C respectively) for cells adjacent to the heterotypic boundary (D) Average orientation 〈*O*〉 as a function of the magnitude of the heterotypic interfacial tension *σ* for the Voronoi (green) and vertex (magenta) models. Error bars represent the standard error with respect to each ensemble.

### 3.2 Dynamic differences between models at the boundary

What mechanism is causing this dramatic geometric/structural difference? For orientation to occur on the tissue boundary in Voronoi models, two things must occur; cells must remain on the interface and elongate perpendicular to the interface. In completely confluent simulations with periodic boundary conditions, for cells to elongate perpendicular to the interface, they must reduce their surface area with the interface, and new cells must fill that gap. This means there must initially be a net flow of cells moving from the bulk to the interface to allow the geometry change. We speculate that this might occur if cells are kinetically pinned at the boundary, so that it is easier for them to move to the boundary than leave. The previously discussed cusp-like restoring force at heterotypic interfaces in Voronoi models [57] does pin cells to the boundary. We hypothesize that the magnitude of this restoring force is lower in the vertex model, which allows more frequent rearrangements at the boundary, and prevents the orientation effect.

To test this hypothesis and measure these restoring forces, we perform a new set of simulations where we initialize a completely segregated system of two different cell types. This system is allowed to relax over 10^5^ time steps with thermal fluctuations and then over an additional 10^6^ steps with no fluctuations to reach an energetic equilibrium. Then a single cell is selected and the cell center is displaced by a length *ϵ* (in natural units) in a direction perpendicular to the interface boundary. In the Voronoi model, we define the restoring force *F*_*r*_(*ϵ*) as the resulting force on the cell center (e.g. the gradient of the energy given by Eq 3 with respect to the cell center, evaluated at that value of the displacement), and in the vertex model as the average force on each vertex that comprises the cell (e.g. gradient of the energy given by Eq 3 with respect to each vertex position in that cell, evaluated at that value of the displacement).

As in previous work, we expect that a cusp in the energy will result in a restoring force that scales linearly with the interfacial tension and is independent of the displacement up to a length scale at which the normal Hookean response starts to dominate. Fig 4A shows that both the Voronoi (different shades of green, solid lines) and vertex (different shades of magenta, dashed lines) models exhibit a flat, non-zero plateau over a range of small displacements, demonstrating the both models exhibit a discontinuous restoring force.

**Fig 4:**
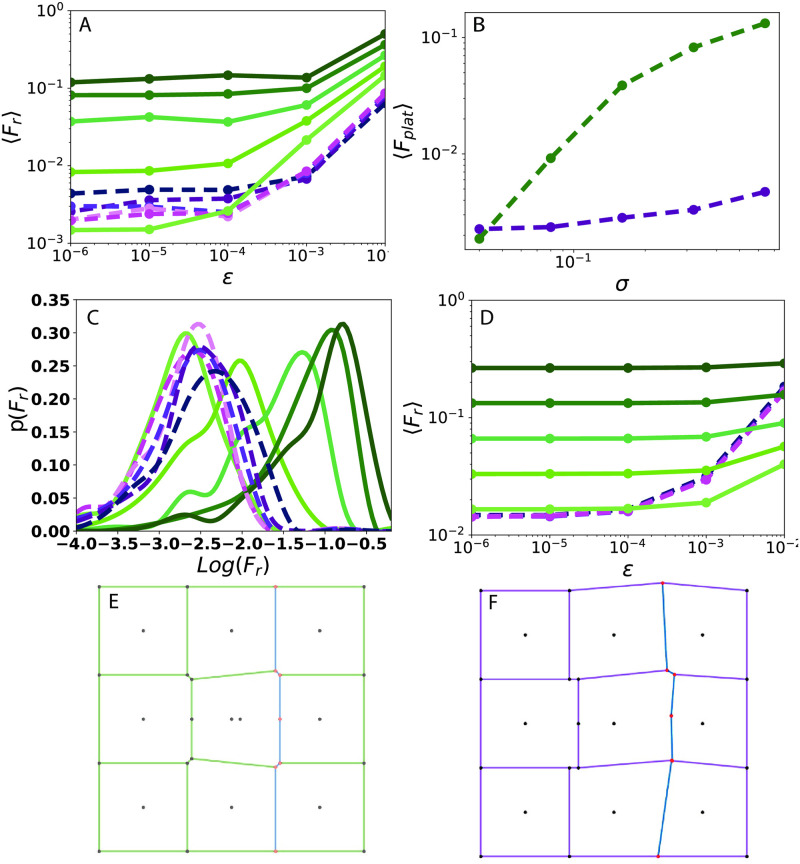
(A) The average cusp-like restoring force for cells, 〈*F*_*r*_〉, being perturbed a distance *ϵ* perpendicular to the heterotypic boundary. The solid green lines are from 3D Voronoi model simulations with *σ* ∈ 0.04, 0.08, 0.16, 0.32, 0.64, with the colorscale ranging from light green at the lowest tensions to dark green at the highest, and the dashed purple lines are from the 3D vertex model with *σ* = 0.04, 0.08, 0.16, 0.32, 0.64 with the color shade ranging from light pink to dark magenta. (B) The plateau values at low tensions extracted from panel (A). (C) The distribution of restoring forces with *ϵ* = 10^−4^ for each set of parameters over an ensemble of *N* = 100 systems. In both systems, the forces are Gaussian-distributed around a central peak (Shapiro-Wilk test with P-values between 1*e*–5 and 1*e*–15), with colorscale the same as in panel (A). (D) Numerical simulations of the restoring force generated by perturbing a single Voronoi cell in 9 cell configuration in 2 dimensions. (E) Schematic diagram illustrating cell boundaries after a perturbation of *ϵ* = 10^−1^ to the cell center of a Voronoi (E, green) cell. (F) Schematic diagram illustrating cell boundaries after adding a random displacement of magnitude *ϵ* = 10^−1^ to each vertex of the center cell. In both (E,F), the blue lines represent the heterotypic interface with increased interfacial tension, and the red dots highlight positions of the vertices along the interface.

The different shades in Fig 4A correspond to different magnitudes of the interfacial tension. For the Voronoi systems, the lowest tension corresponds to the lightest green, while the highest tension correspond to the darkest green, and for the vertex systems, the lowest tension is the lightest pink while the highest tension is dark magenta. This figures shows that the magnitude of this restoring force increases significantly faster as function of the magnitude of the interfacial tension in the Voronoi model, as seen in Fig 4B. This is consistent with the 2D results [31] for the Voronoi model. In contrast, the plateau value in the vertex model is much less sensitive to heterotypic tension magnitude. This results in an order of magnitude difference in restoring force for moderate values of interfacial tension that are approximately 10% higher than the average homotypic tension, and suggests that something different is going on in the vertex model.

For a fixed displacement value *ϵ* = 10^−4^, the distribution of forces over an ensemble of *N* = 100 simulations in Fig 4C shows an approximately normal distribution for both models, with a goodness of fit quantified by a Shapiro-Wilk test with P-values between 10^−5^ and 10^−15^. This suggests that the variation in average restoring force between the two models is not from outlier behavior in either model, but due to a systematic difference in boundary behavior. We hypothesize that the difference in restoring force is due to the extra degrees of freedom in the vertex model that allow fluctuations at the interface to overcome the energetic barriers that protect fourfold coordinated vertices at the interface.

To make this hypothesis more concrete, we construct a simple 9-cell numerical toy model in 2 dimensions, with a geometry shown in Fig 4E and 4F. First, we perturb a single Voronoi cell a displacement *ϵ* perpendicular to the heterotypic interface, Fig 4E, and calculate the resulting force, replicating the work done in Ref [31]. Then, to capture the variability in accessible states in the vertex model, we look at the same initial 9-cell configuration but randomly perturb the vertices on the interface with a magnitude 10^−3^, as seen in Fig 4F. All the vertices of the center cell are displaced by *ϵ* perpendicular to the interface and the resulting restoring force is recorded. We average the restoring force over an ensemble of *N* = 1000 trials. Comparing the 2D toy model in Fig 4D to the full 3D simulations in Fig 4A reveals substantial similarities, suggesting the 2D toy system is capturing the important features.

In the toy 2D vertex model, we can directly show that the insensitivity to heterotypic interfacial tension *σ* arises for a different reason compared to the Voronoi model. In the vertex model, the area and perimeter term contributions to the the restoring force are much larger than contributions from the heterotypic interfacial tension contributions over a wide range of heterotypic interfacial tension values *σ* ≤ 15. This is because in vertex models, unlike Voronoi models, it is no longer the breaking of a four-fold vertex into two three-fold vertices that generates the cusp. In fact, perfect four-fold coordinated vertices have no cusp when perturbed, as shown by the green line in Fig 5B.

**Fig 5:**
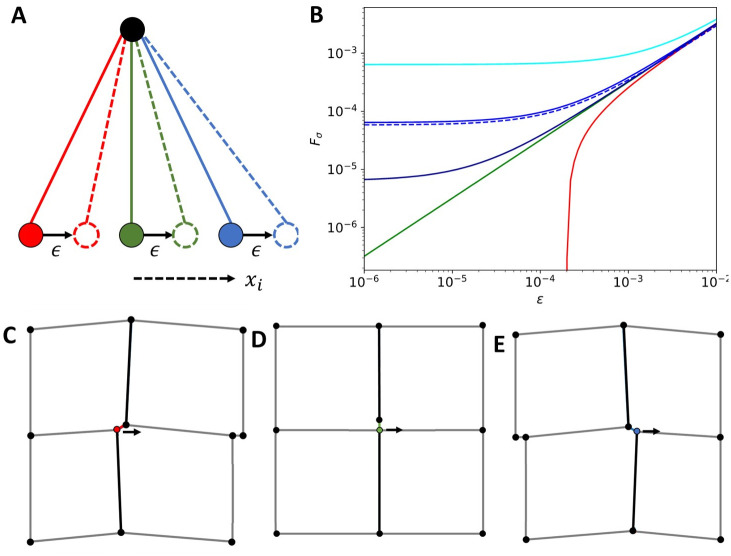
(A) Schematic of perturbations to the *x*–component of the initial position *x*_*i*_ of a single vertex relative to a putative four-fold coordinated vertex on the boundary. The green line represents a perfect four-fold vertex where *x*_*i*_ is zero, the blue represents a coordinate that has a positive *x*_*i*_ towards the interface and the red represents a vertex that is further from the interface *x*_*i*_ negative. (B) Analytic restoring force (Eq 7) due to the interfacial tension alone due to the perturbations of vertices on the interface. The solid lines represent intial perturbations of the moving cell’s vertex a distance *x*_*i*_ = {−10^−4^, 0, 10^−5^, 10^−4^, 10^−3^} colored {red, green, dark blue, blue, cyan} respectively with *y*_*i*_ = 1. The dashed blue line represents a perturbation parallel to the interface such that {*x*_*i*_, *y*_*i*_} = {10^−4^, 1.1}. (C-E) Schematic diagrams of how the larger-scale cellular structure changes when a nearly four-fold coordinated vertex is perturbed as shown in (A).

Instead, the cusps are generated by geometric nonlinearities introduced when a *nearly* four-fold coordinated vertex at the end of a long edge is displaced nearly perpendicularly to that edge, as highlighted by the red and blue lines in the schematic in Fig 5A. Fig 5 panels (C-E) illustrate how these changes to a single vertex that is nearly four-fold coordinated alter the geometry of nearby cells.

To demonstrate this effect, we use coordinates where the stationary boundary point corresponding to a putative perfect four-fold vertex is at {0, 0} and the actual vertex is initially shifted to a position {*x*_*i*_, *y*_*i*_} from this origin. We write an analytic expression for the restoring force when this vertex is then displaced a distance *ϵ* from its initial position in the positive *x*-direction, as shown in Fig 5A:

$$\begin{array}{c} F_{\sigma }=\frac{\sigma }{\epsilon }\left(\sqrt{({\left(x_{i}+\epsilon \right)}^{2}+y_{i}^{2}}-\sqrt{x_{i}^{2}+y_{i}^{2}}\right). \end{array}$$ (7)

This equation confirms that in the limit that the initial x-component *x*_*i*_ is zero, the cusp disappears. In this case of a perfect four-fold vertex, the restoring force scales linearly with the displacement into the interface as shown by the green line in Fig 5B. As also shown in panel (B), the restoring force is lower than the 4-fold expectation if the vertex is initially located in the opposite (negative) direction compared to the displacement *ϵ* (red), and higher than the 4-fold case if the vertex is initially located in the same (positive) direction as *ϵ* (blue). For this “blue” case, the magnitude of the restoring force and the size of the plateau are proportional to the component of the initial displacement perpendicular to the interface *x*_*i*_. Parallel displacements of the initial vertex *y*_*i*_ have a higher order effect on the restoring force. This makes it clear that the origin of the cusp for vertex models is simply the non-linear nature of hypotenuses.

In the vertex model with finite temperature, we expect that there will always be such “red” and “blue” fluctuations of the vertex positions, and since the restoring forces in the blue case are much larger than the red case, they will dominate the average. This effect is what gives rise to the finite, small plateau that is largely independent of interfacial tension for the magenta vertex model curves in Fig 4A and 4D).

### 3.3 Comparison to experimental data

We wanted to compare the results for orientation *O* and registration *R* of cells at a heterotypic interface from Voronoi and vertex models to experimental data in the literature. Unfortunately, the data already available in the literature typically contains only one or two representative images of cell geometries, which are not sufficient to reliably calculate averages of these parameters. Therefore, as described in the methods section above, we analyzed new data sets from images of the heterotypic interface between mesoderm and ectoderm in seven developing Xenopus gastrula. The observed field of view typically encompasses 10-25 cells on each side of the interface. A representative image from a confocal z-stack from one gastrula is shown in Fig 6A, and a manual tracing of cell outlines for cells adjacent to the heterotypic boundary is shown in Fig 6B. We then used this data to compute the best-fit ellipse to each cell shape and the center of mass of each cell (shown in Fig 6C), and then used this information to compute the registration and orientation in exactly the same way as for the simulation data. A histogram of all of the registration values and orientation values for all of the cells in all of the embryos is shown in Fig 6D. In Fig 6E we plot the average value of orientation and registration, where the error shown is the standard deviation of these average values across embryos. For comparison, we also shown the average range of these observables found in vertex (magenta) and Voronoi (green) models with heterotypic interfacial tensions between 0.1 and 1.0, which is a very broad estimate of the possible range of values estimated for heterotypic interfacial tensions in embryonic tissues [30, 37]. Fig 6E demonstrates that the cells at this particular type of heterotypic interface are not highly registered, consistent with the vertex models but not with the Voronoi models. They are also slightly oriented perpendicular to the interface, which is consistent with the Voronoi model results and slightly outside the average of what is seen in vertex models. As discussed below, these observations may also be impacted by specific features of the experimental system that are different from the assumptions we made in either model.

**Fig 6:**
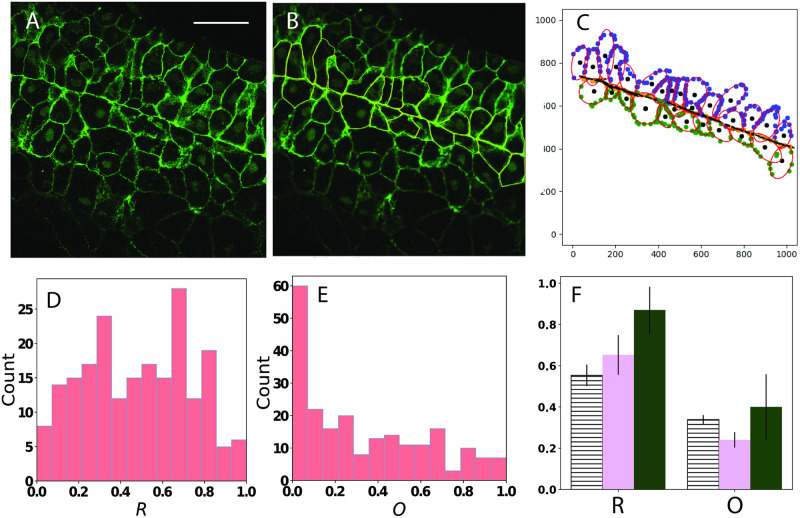
(A) Representative image from a confocal z-stack from one Xenopus gastrula across the ectoderm-mesoderm boundary. Scale bar is 50 *μm*. (B) Example of manual tracing of projected cell shapes from this image. (C) Image illustrating calculation of center of mass (black dot) and best-fit ellipse (red ellipses) for cell shapes across the heterotypic interface. (D-E) Histograms of the average registration *R* for pairs of cells (D), and orientation *O* for all cells (E) in images from seven Xenopus gastrula. (F) Bar plot illustrating the average *O* and *R* in experiments (box with black and white lines), with an error bar representing the standard deviation. For comparison, the average *O* and *R* for the vertex (light magenta) and Voronoi (dark green) simulations across a broad physiological range of heterotypic interfacial tensions (between 0.1 and 1) is also shown, with the error bar representing the max and min values of those observed parameters in simulations for that range of tensions.

## 4 Conclusions and future work

We have investigated differences in structure and dynamics for heterotypic interfaces between two tissue types in the 3D vertex and Voronoi models. Both models share significant similarities in demixing behavior and cell shape on the boundary and the bulk. However, the registration of cell centers (quantified by *R* defined by Eq 4) is significantly smaller in vertex models compared to Voronoi models, and cells on the boundary between tissue types will orient perpendicular to the interface in the 3D Voronoi model but not the vertex model.

Previous work has also characterized the roughness of the heterotypic interface (roughness is typically measured as the width of the interface compared to the length of the interface). Initial simulations of 2D vertex models found interfaces that were unexpectedly rough [37]. Subsequent studies—in a similar parameter regime for the magnitude of the interfacial tension—found interfaces in Voronoi model simulations that were much less rough [31], and that same paper also presented a small data set for 2D vertex models that also had a less-rough interface. An important difference between those two studies is the fluidity of the tissue; the tissue in [37] was solid-like while the tissue in [31] was fluid-like. Due to the limitations in box length for 3D systems from increased computational complexity, we were unable to study a large enough range of box sizes to extract roughness scaling in 3D vertex models in this work. However, the interface image in Fig 2A and the data for cell height fluctuations in Fig 2C demonstrates that typical fluctuations perpendicular to the interface are significantly less than a cell diameter, which is quite different from that seen in [37]. This suggests that surface roughness is not too different between vertex and Voronoi models, and that tissue fluidity may play an important role. This would be an interesting avenue for future work.

In the Voronoi model, the restoring force for perturbations to cells along the boundary is an order of magnitude higher than that of the vertex model for moderate values of interfacial tension. The difference in restoring force arises from the different mechanisms that drive cusps on the boundary; in Voronoi models four-fold vertices must split into pairs of three-fold vertices in response to a perturbation, while in vertex models the cusps are created by subtle geometric nonlinearities and only arise when averaging over fluctuations, resulting in pinning forces that are much weaker. In practice, this difference means that in Voronoi models more cells can be pinned at a heterotypic interface, leading to an orientation effect not seen in vertex models. This indicates that cell shapes at heterotypic boundaries of Voronoi models are a consequence of the representation of the degrees of freedom and not of the underlying energy functional.

We also compared features of the cell shapes found in simulations to those same features in a set of experimental images of a heterotypic interface in Xenopus gastrula, and found that the cells were slightly oriented with their long axis perpendicular to the interface, and that they were not strongly registered. The orientation magnitudes were similar to those seen in a Voronoi model at physiologically relevant values of the heterotypic surface tension, while the registrations were more similar to that seen in vertex models. In our simulations the two tissue types were exactly the same except for a heterotypic interaction, but that is clearly not the case in the experiments. In Xenopus gastrula, ectoderm and mesoderm cells have different projected cross-sectional areas (suggesting different volumes), and also different degrees of elongation (suggesting that the cell target surface areas are different as well). This cell elongation has been noted previously [58], and associated with secreted chemoattractants [58, 59]. All of these effects, as well as a multitude of additional signaling processes, could impact the registration and orientation seen in experiments. Nevertheless, the experimental observations are consistent with our claim that the extreme registrations seen in the Voronoi model—even at very low values of the heterotypic interfacial tension—are not seen in typical experimental data. This makes sense, as we expect real cells are able to independently move their vertices and so their shapes are not governed by the putative location of their center of mass.

As Voronoi models are significantly less computationally intensive and require fewer parameters than vertex models, this suggests that researchers should consider the dynamics and structures they are trying to resolve when choosing how to represent the degrees of freedom in a model. In simulations where dynamics near cell boundaries are not expected to play an important role, both Voronoi and vertex models generate similar mechanical and structural properties. In models that need access to more diverse cell shapes or where researchers are interested in making predictions about cell dynamics near boundaries, the 3D vertex model may be preferable.
